# GPs’ perspectives on patients presenting with endometriosis symptoms: an interview study

**DOI:** 10.3399/BJGPO.2025.0086

**Published:** 2026-02-25

**Authors:** Sólja Petersen, Ulrik Bak Kirk, Maria Pencheri, Sharon Dixon, Rebecca L Mawson, Birgitte Nørgaard, Michael Marcussen

**Affiliations:** 1 Department of Public Health, University of Southern Denmark, Odense, Denmark; 2 Department of Public Health, Aarhus University, Aarhus, Denmark; 3 Research Unit for General Practice, Aarhus University, Aarhus, Denmark; 4 Nuffield Department of Primary Care Health Sciences, University of Oxford, Oxford, UK; 5 Division of Population Health, The University of Sheffield, Sheffield, UK

**Keywords:** general practitioners, endometriosis, primary health care

## Abstract

**Background:**

Endometriosis affects about one in 10 women, yet diagnosis often takes 8–12 years after onset of symptoms. In the Faroe Islands, GPs play a key role in recognising symptoms, managing care, and coordinating referrals to specialists. Therefore, GPs’ perspectives on how challenges to care arise and how care could be improved are crucial in order to develop effective interventions for change.

**Aim:**

To investigate how GPs in the Faroe Islands experience encounters with patients presenting with endometriosis symptoms.

**Design & setting:**

A qualitative interview study was undertaken with six GPs from various practices across the Faroe Islands.

**Method:**

This study conducted semi-structured individual interviews and analysed participants’ responses. Systematic text condensation, inspired by Malterud’s thematic analysis, was used to explore GPs’ perspectives on their interactions with patients presenting with endometriosis symptoms.

**Results:**

Within the constraints of the Faroese healthcare system, many GPs reported difficulties collaborating with gynaecologists on treatment protocols. Limited referral pathways often led to challenges, particularly when referrals were declined or when patients were returned to general practice without clear diagnosis or management plans. GPs expressed frustration with the limited treatment options available in primary care and a sense of being professionally constrained. They emphasised the need for specialist involvement and highlighted the importance of building long-term relationships with patients offering holistic care, managing expectations early, and maintaining continuity of care.

**Conclusion:**

This study highlights the difficulties GPs face when managing and referring patients with suspected endometriosis. It is crucial to enhance collaboration with specialists and improve referral protocols to optimise patients’ care and outcomes.

## How this fits in

This study examines the referral barriers GPs face in managing endometriosis in the Faroe Islands. It highlights the importance of early patient expectation management and strong GP–patient relationships. The findings advocate for stronger collaboration with gynaecologists to enhance quality and continuity of care. This is the first research article to explore endometriosis management in the Faroe islands and aims to evidence the challenges faced by those with the such a common yet often debhilitating condition.

## Introduction

Endometriosis is a condition that affects approximately one in 10 women,^1^ equating to around 190 million women worldwide.^2^ Endometriosis is defined as a chronic condition in which tissue resembling the endometrium is found outside the uterus.^3^ However, it presents with diverse and complex symptoms, including chronic pelvic pain, painful intercourse, heavy bleeding, menstrual pain, and infertility.^4,5^ Its symptoms can vary widely and fluctuate in severity between individuals and over time. Its symptoms are non-specific and may overlap with other common conditions, such as irritable bowel syndrome, bladder symptoms, fatigue, and chronic pelvic pain.^6–9^ While endometriosis is highly prevalent, studies have revealed an average diagnostic delay of 8–12 years between symptom onset and confirmed diagnosis.^10,11^

In Denmark, GPs are the primary contact in the publicly funded healthcare system, ensuring free and equal access for all residents.^12,13^ Similarly, in the Faroe Islands, Faroese GPs serve as gatekeepers to secondary health care. Residents access gynaecological care through referral from their GPs to the National Hospital in Tórshavn, the largest medical facility in the Faroe Islands.^14^

The existing literature demonstrates that GPs encounter many challenges in both diagnosing and managing endometriosis, primarily owing to time constraints and limited opportunities for thorough examinations.^6,7^ Additional factors contributing to diagnostic delays for endometriosis include uncertainty about normal versus abnormal menstrual pain, patients feeling dismissed by healthcare professionals, and patients delaying seeking medical help. These factors collectively hinder timely diagnosis.^15–17^

From evaluating symptoms to establishing a diagnosis and then onward care, GPs play a crucial role in managing endometriosis, as they are expected to have a strong familiarity with their patients and a comprehensive understanding of each patient’s illness and treatment options. Therefore, GPs serve as the professionals primarily responsible for patient care, acting as gatekeepers and coordinators for specialised hospital services and municipal resources.^12,18^ To our knowledge, GPs’ perceptions of managing patients with suspected endometriosis have been inadequately explored. This study aims to enhance understanding of the context in which such care is delivered. Women with endometriosis often report challenging healthcare experiences, including diagnostic delays and feelings of dismissal.^19^ Addressing these issues is central to the objectives of this research. By investigating GPs’ perspectives on care delivery, we seek to identify opportunities to improve management and support for this patient population.

## Method

This study is reported following the Consolidated Criteria for Reporting Qualitative Studies (COREQ), a 32-item checklist for interviews and focus groups.^20^

### Study design

This study used a qualitative design with semi-structured individual interviews to explore GPs’ perceptions of their encounters with patients presenting with endometriosis symptoms. It thematically analysed participants’ responses using Malterud’s systematic text condensation method.^21^

### Participants and recruitment

All GPs were recruited using snowball sampling, which involved email outreach to a representative of the exclusive healthcare insurance provider in the Faroe Islands and direct contact with the chairman of the Faroe Medical Association. These key gatekeepers were crucial in accessing GPs throughout the Faroe Islands by distributing the information letter to their 36 members. The final sample included six GPs working in different settings, with varying practice sizes and multiple geographical areas. To ensure confidentiality and anonymity, further details are not disclosed. There was no formal patient or public involvement in the conduct of this study. However, patients were involved in developing the interview guide.

### Study context: endometriosis in Faroe Islands general practice

As of 2025, 36 GPs serve a population of approximately 55 000 residents, of which 11 000 are Faroese women of childbearing age.^22,23^

### Researcher characteristics and reflexivity

The interviewer (STP) is a female researcher with personal and professional experience in endometriosis. The research team included diverse expertise in health sciences, including GPs. While their preconceptions influenced the research process, open-ended questions ensured consistency and allowed participants to express their views, emphasising the importance of diversity in qualitative research.^24^

### Interview guide

A semi-structured interview guide was prepared, inspired by Malterud’s approach,^21^ aiming to maintain a consistent focus on the subject matter.^25^ The first author (STP) developed and refined this guide in collaboration with the last author (MM). The guide was pilot tested by both the first and last authors (STP and MM) in two interviews, which led to minor revisions, including rephrasing questions for clarity and modifying their sequence to enhance flow. These pilot interviews are not included in the final sample of interviews. The main open-ended questions asked during the interviews were:

- What has been your experience of supporting or evaluating patients reporting/describing symptoms of endometriosis?
- What has been your experience of managing these patients?
- Could you expand on this with any examples from your work?

### Data collection

The first author (STP), who is Faroese and fluent in both Faroese and Danish, conducted six individual interviews: five in person at the GP practices and one online using Zoom. Five interviews were conducted in Faroese and one in Danish. All interviews conducted in Faroese were subsequently translated into Danish by the first author (STP). The interviews took place between March 2024 and April 2024, lasted 35–40 minutes, and were audiorecorded and transcribed verbatim by the first author (STP). None of the participants were previously known to the interviewer.

### Analysis

The data were thematically analysed in four steps, as described by Malterud.^26^ This approach, rooted in Giorgi’s phenomenology,^27^ allowed participants to share their lived experiences, which is central to the study’s aims. The four steps were as follows: (1) Possible themes*:* we created a general overview, identifying preliminary themes relevant to the research question; (2) De-contextualisation: we identified and organised data elements that elucidated the research question by systematically reviewing the transcripts line by line to extract meaningful units; (3) Condensation: we reduced the empirical data to a decontextualised selection of meaningful units, which was then sorted into thematic code groups across participants; (4) Synthesis: we discussed the initial analysis again and further refined and developed the themes. Finally, we reconceptualised the data by developing descriptions, concepts, and narratives that elucidated the research question.^26^

Two authors (STP and MM) independently analysed the data using NVivo (version 14) software to identify patterns and sub-themes, then discussed their findings with two co-authors (GPs and qualitative researchers) to reach a consensus.^28^ Any discrepancies in interpretation were resolved through discussion within the research team until agreement was achieved.

An example of the thematic analysis process is presented in Table 1.

**Table 1.** The four steps of the thematic analysis exemplified

Step 1. Possible themes: overall impression	Step 2. De-contextualisation: from themes to codes	Step 3. Condensation: from codes to meaning	Step 4. Synthesis: from condensation to description and concepts
**Managing expectations**	*‘I believe it can be frustrating when expectations don’t align. For instance, a patient might read about endometriosis and anticipate being seen by a gynaecologist ...’*	Having that conversation early in the process is essential.	The GPs emphasised the critical importance of managing patient expectations for those exhibiting endometriosis symptoms. Four of the five interviewed GPs highlighted the need to manage patient expectations early in the process.
**Gatekeeper function**	‘*This creates a situation where gynaecologists may prefer that we* [the GPs] *refrain from referring patients with endometriosis unless there are considerations for surgery or fertility, as if the patient must be planning to have a child ...’You often feel helpless in practice because when you refer a patient to the gynaecologist and they are told that everything looks normal and that “there’s nothing wrong with you”, it can be disheartening. After all, the specialists are the experts.’*	Experiencing a sense of helplessness while bearing the responsibility alone. It can be challenging to navigate a system where referrals are limited to one place.	By validating and acknowledging the patient’s concerns, GPs find that it encourages greater receptiveness to further treatments. Many patients have endured a long journey without effective relief for their symptoms. The GPs noted that those with endometriosis and chronic pain often struggle to be taken seriously, which can lead them to develop additional psychosocial disorders if their long-standing pain is not addressed.

### Ethical considerations

This study complied with ethical principles for medical research described in the Declaration of Helsinki.^29,30^ Before initiation, this study was approved by the institutional review board at the University of Southern Denmark (ID:12.166). All data were managed in compliance with the European General Data Protection Regulations.^31^ Before inclusion, GPs were thoroughly informed about the study’s objectives through verbal and written communication and provided written informed consent. They were also informed that their participation was voluntary, that they could withdraw their consent at any time without consequence, and that their statements would be treated confidentially and anonymised. According to Danish legislation, no further approval was required.^32^

## Results

The following four key themes emerged from the analysis: managing expectations; gatekeeper function; patient rights; and individual treatment. The findings are organised around these themes and further illustrated with relevant quotes from participants to provide context and depth.

### Managing expectations

All GPs reported that patients often hold high and sometimes unrealistic expectations regarding both treatment options and the possibility of urgent referrals to specialised care. All GPs emphasised the importance of managing these hopes early through expectation reconciliation. This process was seen as essential for maintaining trustful GP–patient relationships, as patients often anticipated solutions that exceeded the GP’s capacity to deliver. Several GPs described their role in addressing patient expectations based on prior experiences with referrals or care-seeking behaviour. They observed that patients frequently held unrealistic beliefs about gynaecologists’ ability to provide immediate solutions for their symptoms and pain, underscoring the critical role of clear communication and expectation management in consultations with these patients:

*‘… The patients believe the gynaecologist can resolve their issues and make their pain disappear, even when that isn’t possible.’* (GP3)

All GPs stressed that managing patient expectations early improves both treatment outcomes and the GP–patient relationship:

*‘It’s much easier for me to address things early on in the process. Once they have some initial information, they can come back to me with questions. This makes it simpler to establish a strong GP–patient relationship. I want them to feel confident in sharing their thoughts with me, and I make sure they trust that I take their concerns seriously and know what I’m doing. Building that trust starts right from the beginning.’* (GP6)

All GPs voiced frustration over the challenges of managing patient expectations, particularly when patients often arrive with information about endometriosis and anticipate immediate referrals to a gynaecologist. However, they explained that certain procedural steps must first be completed within general practice before a referral can be made. This misunderstanding can lead to patient dissatisfaction, as they may feel delayed in accessing secondary care despite the GP’s efforts to provide supportive guidance. To address this, all GPs emphasised the importance of managing expectations early, noting that the referral pathway is complex and involves several steps before a referral to a specialist can be made. By proactively addressing these expectations, all GPs felt better equipped to provide effective care and manage patient concerns. Several GPs noted that this proactive communication helped patients understand what to expect when contacting their GP, thereby reducing their frustration and fostering greater collaboration and trust in the GP–patient relationship.

### Gatekeeper function

All GPs frequently encountered patients who strongly requested referrals to a gynaecologist; however, gynaecologists had previously advised all GPs to limit such referrals for endometriosis owing to extensive waiting lists, asking them to prioritise other patient groups. This situation puts all GPs under pressure from both patients and gynaecologists, creating difficult dilemmas for them because managing endometriosis often extends beyond their expertise. These cases underscore the challenges all GPs face in balancing patient needs with systemic constraints:

*‘The gynaecologist cannot take any action until the initial steps are addressed here in general practice. However, patients often don’t understand this and insist on seeing a gynaecologist right away. I’m trying to manage resources effectively, which makes this a significant challenge.’* (GP1)

The gynaecologists advised all GPs to follow standardised treatment protocols, including the use of pain relief medications (analgesics) and hormone therapy, before considering referrals. In order to maintain patient trust, all GPs prioritised building strong GP–patient relationships through education, ensuring that all treatment options available in general practice are thoroughly discussed. All GPs emphasised the importance of adopting a holistic approach, recognising that some patients might also experience psychosocial challenges. Furthermore, all GPs acknowledged the difficulty in managing symptoms that were not always explainable or validated by diagnostic tests:

*‘There are two main aspects to consider. The first is the professional side, where we focus on diagnosis. The second aspect involves the psychosocial factors, which often play a significant role. This includes how the patient manages their education, relationships, and other life circumstances. Sometimes, these psychosocial factors can be just as impactful and occupy as much attention as the illness itself.’* (GP3)

Consequently, all GPs stressed the need to approach these interactions with empathy, affirmation, and appreciation, fostering a supportive and understanding environment for this patient group:

*‘Often, it is beneficial for her to feel heard and to have a safe space to come to without experiencing additional distress. It is crucial to convey that I genuinely believe her.’* (GP2)

All GPs emphasised validating patients’ experiences, even when the cause of pain is unclear, recognising that symptoms affect physical, social, and mental wellbeing, and require holistic discussions.

All GPs described challenges when patients’ referrals to the gynaecological department for endometriosis investigations are denied, complicating their ability to explore alternative treatment options within primary care. Additionally, when gynaecological assessments reveal no abnormalities, patients are often returned to general practice without further recommendations despite their persistent symptoms. This lack of guidance added to the complexity of managing these cases:

‘*Our greatest challenge is the feeling of being unable to provide the specialised treatment that our patients need. This situation is particularly difficult for GPs, as the responsibility ultimately returns to us.’* (GP4)

When patients’ referrals are denied because their pain is deemed insufficient for further endometriosis investigations or when they are sent back to general practice owing to inconclusive findings, all GPs described a sense of powerlessness and frustration when left to shoulder the full responsibility for managing these patients’ care, against their preference, having tried to seek specialist support:

*‘Our greatest challenge is the feeling of being unable to manage on your own. You seek help from a specialist, only to find out that your request is denied.’* (GP1)

The GPs expressed frustration over the challenges posed by the limited availability of referral options:

*‘It is a challenge that I often do not have a specific place to refer them to, and even when I do make a referral, you often find nothing ... As a result, the situation remains unresolved and lacks follow-up.’* (GP1)

All GPs perceived the lack of referral options as a barrier, leading to helplessness. They relied on specialists without receiving clear guidance and faced uncertainty owing to the absence of protocols for managing endometriosis in general practice:

*‘It is reassuring to have followed established protocols and to transfer responsibility to a specialist, but without clear instructions, it becomes more challenging to assert that I have fulfilled my role and that the next steps are now in your hands. We find ourselves lacking the authority to dictate the process and can only make referrals.’* (GP4)

All GPs recognised the limited capacity of the gynaecology department, where patients with more acute conditions were prioritised over those with endometriosis symptoms. Despite understanding these constraints, they felt a strong responsibility to advocate for their patients:

*‘However, I also understand that they* [gynaecologists] *need to prioritise, which is why we must ensure that we write thorough referrals so that the patients can be appropriately prioritised.’* (GP3)

All GPs acknowledged gynaecologist’s resource constraints but stressed the need to address patients with persistent symptoms, even with normal test results for endometriosis. They felt gynaecologists overlooked the challenges in general practice, which hindered effective management of endometriosis.

### Patient rights

All GPs valued a diagnosis and recognised its value and meaning for patients. However, they also recognised that the same treatment could be offered whether or not the patient was diagnosed with endometriosis. They disagreed on the value of investigations, with some believing the underlying cause of pain was irrelevant to treatment, regardless of an endometriosis diagnosis:

*‘I have spoken with the gynaecologists, who have stated that they are not doing anything differently from what we* [GPs] *are doing. It does not alter the treatment or the prognosis. Therefore, I do not always refer them further. Sometimes, I initiate treatment with oral contraceptives, an intrauterine device, or another approach before referring them.’* (GP1)

All GPs observed that if the prescribed medication alleviated a patient’s pain, they often inferred a likely diagnosis of endometriosis and proceeded to manage their treatment accordingly:

*‘However, when I suggest that taking birth control pills continuously might provide relief, it leads me to strongly suspect that the individual has endometriosis.’* (GP1)

Conversely, some GPs emphasised the need to prioritise awareness of endometriosis and its potential long-term impact on patients’ quality of life:

*‘Fortunately, there has been increased awareness surrounding endometriosis, particularly among young people. When patients themselves advocate for their symptoms, it becomes easier to identify those who come in with abdominal pain, as this could be indicative of various conditions. Therefore, this heightened awareness can facilitate earlier detection, potentially preventing the condition from worsening.’* (GP4)

However, all GPs experienced a notable deficiency in information regarding managing endometriosis within primary care. The existing guidelines were either inadequate or entirely lacking, necessitating that GPs independently seek out subjective resources to inform their practice:

*‘I was sitting last week looking up some recommendations about endometriosis. I found that there is a significant absence of clinical guidelines in the Faroe Islands, which complicates the management of this condition …’* (GP4)

Some GPs highlighted that a normal gynaecological examination did not necessarily rule out endometriosis. In such cases, they continued to manage care for patients suspected of having endometriosis, which proved particularly challenging when they disagreed with gynaecologists’ evaluations:

*‘If the gynaecologists reject the diagnosis but you still believe it is endometriosis, then I would continue to consider it as such.’* (GP3)

All GPs voiced concerns about the potential consequences for patients’ access to necessary treatment if they relied solely on gynaecologists’ conclusions, particularly when no abnormalities were detected during examinations.

### Individual treatment

Several GPs reported having to adjust their treatment approaches to align with the limited resources available in general practice:

*‘… if medications ie, Brufen* [ibuprofen] *and others hadn’t been effective, I would have explored other options, such as referring the patient to a psychologist, and looked into alternative ways to support them in managing their condition and improving their quality of life.’* (GP2)

Collaboration with psychologists and physiotherapists is key to developing comprehensive treatment plans. All GPs stressed a holistic approach, recognising endometriosis’ impact on mental health and social wellbeing, and included referrals to these professionals as an integral part of care.

All GPs emphasised the importance of addressing patients’ psychological symptoms alongside physical ones, recognising that chronic pain often led to depression and other mental health issues. They also noted the impact of endometriosis-related pain on employment and highlighted the need for referrals to municipal services, such as job placement assistance, when patients’ work capacity was reduced.

The GPs recognised their responsibility for patient care but emphasised the need to consider the patients’ resources and capacity for treatment. With limited resources, GPs adjusted their communication and strategies, exploring alternative treatment options:

*‘There are times when I feel that if I can’t alleviate her pain and discomfort, she may perceive that I’m not doing everything possible for her. I imagine that when someone is struggling so much, they might be left with the impression that no one is truly helping them.’* (GP2)

All GPs emphasised the need for a supportive approach when patients faced severe pain with no effective treatments. Reassuring patients and allocating extra consultation time helped manage expectations, plan treatments, and build trust in the GP–patient relationship. One GP highlighted that proper preparation was essential for meaningful care:

*‘I have to remember to prepare them properly to inform them properly, right from the start. So that you don’t send them out into something, like where I don’t actually remember to take care of all the things that I want to, and that’s what happens if we’re too busy.’* (GP3)

All GPs acknowledged that these patients required additional time and resources to manage their treatment effectively. Despite the constraints of general practice, the GPs expressed a strong commitment to providing the best possible care within available resources.

## Discussion

### Summary

This study highlighted the challenges GPs face within the Faroe Islands’ geographically constrained system when managing patients with endometriosis, particularly in collaborating with gynaecologists when referrals were declined or unclear. All GPs stressed the importance of setting patient expectations early, building strong relationships, and adopting a holistic care approach, as patients often have evolving symptoms and needs. The study also revealed a resource gap in general practice, limiting GPs’ ability to fully address patient needs. Bridging this gap requires additional time for personalised treatment plans and collaboration with other professionals such as psychologists and physiotherapists. A multidisciplinary approach is essential to support patients’ employment and provide comprehensive care for managing endometriosis symptoms.

### Strengths and limitations

A key strength of our study is the diverse experiences of GPs, providing valuable insights into managing endometriosis and addressing relevant themes. To ensure dependability, all interviews were conducted by the first author (STP) using a semi-structured interview guide, with detailed documentation throughout. The study’s reliability was further enhanced by close collaboration within the research team during data analysis and discussions.

The interviewer (STP) was mindful of the potential influence of her personal and professional engagement with endometriosis. This risk was minimised using Malterud’s systematic text condensation during the analysis. This approach emphasises reflexivity and bracketing to reduce subjective influence and strengthen the study’s trustworthiness.^33^

The inclusion of multiple participant quotations in the Results section further enhances transparency and supports the credibility of the findings. Additionally, an independent review by co-authors reinforced the rigour and reliability of the analysis.^34^ Also, the involvement of co-authors with general practice expertise added credibility, as much of the existing literature lacks input from professionals in the field. Our study’s credibility was also bolstered by obtaining perspectives from multiple GPs.^35^ Moreover, our study focused on the primary healthcare sector, which has received relatively little attention in the existing literature, where the focus has predominantly been on the secondary healthcare sector.^36^

Our study had some limitations. First, owing to the small GP population in the Faroe Islands, anonymising participants restricted our ability to share details about personal experiences and geographical differences. Second, recruitment was challenged, with only six GPs participating. While a larger sample could yield different results, these six represented nearly one-fifth of the total GP population, which is considered acceptable. Owing to confidentiality concerns and participant preferences, detailed location data could not be disclosed, despite two attempts to obtain consent. All participating GPs explicitly declined to share this information, citing concerns that even limited details might compromise their anonymity. Nonetheless, it is important to note that participants represented a range of practice types and geographic regions. Despite this, their diverse perspectives strengthen the relevance of our findings to similar healthcare settings.

### Comparison with existing literature

A key finding of our study was the GPs’ recognition of the complex and multifaceted symptoms experienced by patients with endometriosis and the significant support these patients require. While the GPs acknowledged their primary responsibility for managing treatment, they identified a lack of the necessary skills and resources to provide effective care within general practice settings. These findings are consistent with those of Frayne *et al* ^6^ who highlighted the challenges GPs face in delivering consistent care owing to the lack of specific diagnostic tools and clinical guidelines. Despite these limitations, all GPs stated a strong commitment to supporting their patients, even when the challenges exceeded their professional capacity.

All GPs expressed feeling powerless when standard treatments failed to relieve patients’ symptoms, often leading to increased risk of depression, anxiety, and stress. Casalechi *et al* linked chronic pelvic pain in endometriosis to a higher prevalence of psychological conditions.^37^ Despite recognising the need for a holistic approach, GPs cited limited consultation time as a major barrier, aligning with the Dixon *et al* findings on time constraints and obstacles.^38^ Similarly, Grundstrom *et al* emphasised addressing both the mental and physical aspects of patients’ pain.^39^ The GPs often scheduled additional consultations and follow-up appointments to mitigate the challenge of time constraints. This approach is consistent with the findings of Frayne *et al*, who noted that the complexity of endometriosis symptoms frequently required longer or more frequent visits to meet patients’ needs adequately.^6^

Some GPs reported that increased media attention positively impacted their clinical practice by raising awareness of endometriosis during patient evaluations and improving the referral process to gynaecological departments. This heightened awareness encouraged patients to take a more active role in their care^40^ and a study from Australia highlighted how social media even empowered patients to become active participants in managing their health.^41^ However, the UK media’s one-sided portrayal of GPs as responsible for diagnostic delays in endometriosis oversimplifies a multifaceted issue and unjustly assigns blame to a single group of clinicians.^36,38^ This narrative is echoed in specialist endometriosis literature, where GPs are often described as a bottleneck in the diagnostic pathway.^11,42^

Our study highlights challenges in GP–gynaecology collaboration, with GPs reporting frequent denial of endometriosis referrals, as gynaecologists argued treatment in both sectoral settings were similar. This mirrors findings from the Netherlands, where GPs also faced referral difficulties.^43^ GPs expressed concern over long waiting times for gynaecological appointments, delaying diagnosis and treatment.^44^ Despite this, referrals were typically seen as a last resort, after exhausting other treatment options. This aligns with Ellis *et al*’s findings, where GPs in New Zealand explored alternative treatments, such as exercise, diet, and physiotherapy, before referring patients.^45^

All GPs often received patients back without clear explanations for their pain, as gynaecologists prioritised more acute cases over endometriosis. This may lead GPs to view follow-up consultations as less impactful, given denied referrals or long waiting lists. The issue is not a lack of knowledge about endometriosis among GPs, as previous studies suggest,^16^ but rather the limited management options and access to specialist care when standard approaches and guidelines are insufficient.^36^ Some of our findings are contextually situated to primary care within remote or insular communities; for example, the relative lack of choice of referral options that intersected with the challenge of managing deferred or rejected referrals. However, we note that there are also many points of resonance with findings described in other settings; for example, striving to offer holistic support and co-ordinating care between services, resources, and treatments. While we cannot, and do not, suggest our results are generalisable, we consider that our study findings may be applicable in other settings, both nationally and gloabally .^46,47^

Overall, our study emphasised that all GPs took on considerable responsibility in managing patients with endometriosis. They focused on setting realistic expectations early, embraced a holistic approach to care, and prioritised building and maintaining strong GP–patient relationships despite facing systemic challenges.

### Implications for research and practice

All GPs reported challenges in collaborating with gynaecology departments, often feeling isolated and unable to access specialist support. They emphasised the importance of setting patients’ expectations early and building strong GP–patient relationships. Our findings also highlighted the need for personalised treatment plans addressing both the physical and psychosocial challenges. Given the impact of chronic pain on mental health and social wellbeing, GPs should consider referrals to psychologists. This aligns with existing research on mental health and social effects of endometriosis.

Ongoing awareness campaigns are recommended to highlight endometriosis as a major health issue. An example of this is the successful co-created social media campaign in Denmark.^40^ Further research is warranted to better elucidate GPs’ perceptions of their role in the management of endometriosis. Expanding the sample size in future studies would improve the generalisability of the findings.^48^ Additionally, incorporating patients’ perspectives on endometriosis care in the Faroe Islands — an area that remains underexplored — would provide critical insights. Understanding these perspectives is essential for a more comprehensive view of the diagnostic and treatment challenges associated with the condition.^48,49^

Since our study focused exclusively on GPs’ experiences, future research should also investigate the perspectives of gynaecologists in managing patients with endometriosis symptoms. Such research would provide a more comprehensive understanding of the challenges and facilitate stronger collaboration between GPs and gynaecologists, ultimately improving patient care.

## Data

No datasets were created as part of this project.

## References

[1] Rogers PAW, D’Hooghe TM, Fazleabas A (2009). Priorities for endometriosis research: recommendations from an international consensus workshop. Reprod Sci.

[2] World Heatlh Organization (WHO) (2023). Endometriosis. https://www.who.int/news-room/fact-sheets/detail/endometriosis.

[3] Farquhar CM (2000). Extracts from the clinical evidence. Endometriosis. BMJ.

[4] Pugsley Z, Ballard K (2007). Management of endometriosis in general practice: the pathway to diagnosis. Br J Gen Pract.

[5] Gete DG, Doust J, Mortlock S (2023). Impact of endometriosis on women’s health-related quality of life: a national prospective cohort study. Maturitas.

[6] Frayne J, Milroy T, Simonis M, Lam A (2023). Challenges in diagnosing and managing endometriosis in general practice: a Western Australian qualitative study. Aust J Gen Pract.

[7] Johnson NP, Hummelshoj L, World Endometriosis Society Montpellier Consortium (2013). Consensus on current management of endometriosis. Hum Reprod.

[8] Allaire C, Bedaiwy MA, Yong PJ (2023). Diagnosis and management of endometriosis. CMAJ.

[9] Dunselman GAJ, Vermeulen N, Becker C (2014). ESHRE guideline: management of women with endometriosis. Hum Reprod.

[10] Ducarme G, Planche L, Lebœuf A (2021). Screening and management of endometriosis by primary care physicians. Gynecol Obstet Fertil Senol.

[11] Hudelist G, Fritzer N, Thomas A (2012). Diagnostic delay for endometriosis in Austria and Germany: causes and possible consequences. Hum Reprod.

[12] Pedersen KM, Andersen JS, Søndergaard J (2012). General practice and primary health care in Denmark. J Am Board Fam Med.

[13] Olejaz M, Juul Nielsen A, Rudkjøbing A (2012). Denmark health system review. Health Syst Transit.

[14] Ministry of Health (2023). The Faroese Hospital Service.

[15] Ballard K, Lowton K, Wright J (2006). What’s the delay? A qualitative study of women’s experiences of reaching a diagnosis of endometriosis. Fertil Steril.

[16] Roullier C, Sanguin S, Parent C (2021). General practitioners and endometriosis: level of knowledge and the impact of training. J Gynecol Obstet Hum Reprod.

[17] Nnoaham KE, Hummelshoj L, Webster P (2011). Impact of endometriosis on quality of life and work productivity: a multicenter study across ten countries. Fertil Steril.

[18] Lee K, Titlestad SB, Nørgaard B (2022). Patient perspectives on the management of COPD and type 2 diabetes in general practice: an interview study. BMC Prim Care.

[19] Brauer L, de Cruppé W, Geraedts M (2025). Take me seriously: a qualitative interview study exploring healthcare experiences of endometriosis patients. PLoS One.

[20] Peditto K (2018). Reporting qualitative research: standards, challenges, and implications for health design. HERD.

[21] Malterud K (2011). Kvalitative metoder i medisinsk forskning: en innføring [Qualitative methods in medical research: an introduction]..

[22] Weihe P (2022). Sundhedsvæsnet på Færøerne er lig det danske — dog med undtagelse [The health care system in the Faroe Islands is similar to the Danish one - although with the exception of 2022].

[23] Statistics Faroe Islands (2024). Population. https://hagstova.fo/fo/folk/folkatal/folkatal.

[24] O’Brien BC, Harris IB, Beckman TJ (2014). Standards for reporting qualitative research: a synthesis of recommendations. Acad Med.

[25] Brinkmann S, Tanggaard L (2020). Qualitative methods: a basic book.

[26] Malterud K (2012). Systematic text condensation: a strategy for qualitative analysis. Scand J Public Health.

[27] Beck CT (2021). Introduction to Phenomenology: Focus on Methodology.

[28] Bazeley BCP, Jackson K (2015). Qualitative data analysis with NVivo (2nd ed): (2013). London: Sage. Qual Res Psychol.

[29] World Medical Association (2013). World Medical Association Declaration of Helsinki: Ethical Principles for Medical Research Involving Human Subjects. JAMA.

[30] Health Research Authority (2024). Declaration of Helsinki revisions: our response to the consultation. https://www.hra.nhs.uk/about-us/news-updates/declaration-helsinki/#:~:text=Last%20updated%20on%208%20Jul%202024.%20The%20Declaration%20of%20Helsinki.

[31] Data Protection Act (2018). Act on Supplementary Provisions to the Regulation on the Protection of Natural Persons with regard to the Processing of Personal Data and on the Free Movement of Such Data (Data Protection Act).

[32] Danish Ministry of Justice (2018). The Danish Data Protection Agency.

[33] Malterud K, Siersma VD, Guassora AD (2016). Sample size in qualitative interview studies: guided by information power. Qual Health Res.

[34] Ritter C, Koralesky KE, Saraceni J (2023). Invited review: qualitative research in dairy science — a narrative review. J Dairy Sci.

[35] Ahmed SK (2024). The pillars of trustworthiness in qualitative research. J Med Surg Public Health.

[36] Dixon S, Mawson R, Kirk UB, Horne AW (2024). Endometriosis: time to think differently (and together). Br J Gen Pract.

[37] Casalechi M, Vieira-Lopes M, Quessada MP (2021). Endometriosis and related pelvic pain: association with stress, anxiety and depressive symptoms. Minerva Obstet Gynecol.

[38] Dixon S, McNiven A, Talbot A, Hinton L (2021). Navigating possible endometriosis in primary care: a qualitative study of GP perspectives. Br J Gen Pract.

[39] Grundström H, Kjølhede P, Berterö C, Alehagen S (2016). 'A challenge' — healthcare professionals’ experiences when meeting women with symptoms that might indicate endometriosis. Sex Reprod Healthc.

[40] Stanek DB, Hestbjerg I, Hansen KE (2023). Not 'just a bad period' — the impact of a co-created endometriosis social media health campaign: a mixed methods study. Front Commun.

[41] Adler H, Lewis M, Ng CHM (2024). Social media, endometriosis, and evidence-based information: an analysis of Instagram content. Healthcare (Basel).

[42] House of Commons, Women and Equalities Committee (2024). Women’s reproductive health conditions: first report of session 2024–25. https://committees.parliament.uk/publications/45909/documents/228040/default/.

[43] van der Zanden M, Arens MWJ, Braat DDM (2018). Gynaecologists’ view on diagnostic delay and care performance in endometriosis in the Netherlands. Reprod Biomed Online.

[44] van der Zanden M, Teunissen DAM, van der Woord IW (2020). Barriers and facilitators to the timely diagnosis of endometriosis in primary care in the Netherlands. Fam Pract.

[45] Ellis K, Meador A, Ponnampalam A (2024). Survey of general practitioner perspectives on endometriosis diagnosis, referrals, management and guidelines in New Zealand. Health Expect.

[46] Johansson AM, Söderberg S, Lindberg I (2014). Views of residents of rural areas on accessibility to specialist care through videoconference. Technol Health Care.

[47] Larsen AT, Klausen MB, Højgaard B (2020). Primary health care in the Noric countries: comparative analysis and identiification of challenges.

[48] Wallace LS, Wexler RK, McDougle L (2014). Voices that may not otherwise be heard: a qualitative exploration into the perspectives of primary care patients living with chronic pain. J Pain Res.

[49] Stagno S, Crapanzano K, Schwartz A (2016). Keeping the patient at the center: teaching about elements of patient-centered care. MedEdPORTAL.

